# Gastric Cancer Is the Leading Cause of Death in Italian Adult Patients With Common Variable Immunodeficiency

**DOI:** 10.3389/fimmu.2018.02546

**Published:** 2018-11-05

**Authors:** Federica Pulvirenti, Antonio Pecoraro, Francesco Cinetto, Cinzia Milito, Michele Valente, Enrico Santangeli, Ludovica Crescenzi, Francesca Rizzo, Stefano Tabolli, Giuseppe Spadaro, Carlo Agostini, Isabella Quinti

**Affiliations:** ^1^Department of Molecular Medicine, Sapienza University of Rome, Rome, Italy; ^2^Department of Translational Medical Sciences and Center for Basic and Clinical Immunology Research, University of Naples Federico II, Naples, Italy; ^3^Department of Medicine DIMED, University of Padova, Padova, Italy; ^4^Department of Woman and Child Health, Fondazione Policlinico Universitario A. Gemelli IRCCS, Rome, Italy; ^5^Clinical Epidemiology Unit, IDI-IRCCS, FLMM, Rome, Italy

**Keywords:** common variable immunodeficiency: cancer, gastric cancer, lymphoma, IgA, upper endoscopy, risk, guidelines

## Abstract

An increased prevalence of malignant lymphoma and of gastric cancer has been observed in large cohorts of patients with common variable immunodeficiency (CVID), the most frequently symptomatic primary immunodeficiency. Surveillance strategies for cancers in CVID should be defined based on epidemiological data. Risks and mortality for cancers among 455 Italian patients with CVID were compared to cancer incidence data from the Italian Cancer Registry database. CVID patients showed an increased cancer incidence for all sites combined (Obs = 133, SIR = 2.4; 95%CI = 1.7–3.5), due to an excess of non-Hodgkin lymphoma (Obs = 33, SIR = 14.3; 95%CI = 8.4–22.6) and of gastric cancer (Obs = 25; SIR = 6.4; 95%CI = 3.2–12.5). CVID patients with gastric cancer and lymphoma had a worse survival in comparison to cancer-free CVID (HR: 4.8, 95%CI: 4.2–44.4 and HR: 4.2, 95%CI: 2.8–44.4). Similar to what observed in other series, CVID-associated lymphomas were more likely to be of B cell origin and often occurred at extra-nodal sites. We collected the largest case-series of gastric cancers in CVID subjects. In contrast to other reports, gastric cancer was the leading cause of death in CVID. Standardized mortality ratio indicated a 10.1-fold excess mortality among CVID patients with gastric cancer. CVID developed gastric cancer 15 years earlier than the normative population, but they had a similar overall survival. Only CVID diagnosed at early stage gastric cancer survived >24 months. Stomach histology from upper endoscopy performed before cancer onset showed areas of atrophic gastritis, intestinal metaplasia or dysplasia. CVID patients might progress rapidly to an advanced cancer stage as shown by patients developing a III-IV stage gastric cancer within 1 year from an endoscopy without signs of dysplasia. Based on high rate of mortality due to gastric cancer in Italian CVID patients, we hereby suggest a strategy aimed at early diagnosis, based on regular upper endoscopy and on *Helicobacter pylori* infection treatment, recommending an implementation of national guidelines.

## Introduction

Inherited conditions affecting immune system function are classified as primary immune deficiencies (PID) (1). As the PID life expectancy increased because of improvements in the surveillance, prevention, and treatment, the occurrence of cancer increased (2, 3). In PID, hematological and non-hematological malignancies occur mainly in the fourth to the seventh decades of life while rare case of malignancies are commonly observed in the pediatric population (4). Among PID, an increased prevalence of cancer is recognized in patients affected by common variable immunodeficiency (CVID), the most common symptomatic primary antibody defect. In CVID, the antibody deficiency might derive from decreased diversity of the naive pool, decreased hyper mutation in memory repertoires, an unusual clonal expansion of un-mutated B cells, and from a number of defects in innate and adaptive immune mechanisms (5). Other than sino-pulmonary infections, CVID patients suffer from associated clinical conditions, including autoimmune and inflammatory diseases and neoplasia, mainly lymphoma and gastric cancer (6, 7). Ten years ago, our group described a higher prevalence of lymphoma and gastric carcinomas in the Italian cohort of CVID in comparison to the normative population (8). Five years later, we confirmed this high prevalence rate of lymphoma and gastric carcinomas in a four decades study showing that 21% of adult CVID patients developed cancers. We also observed that deaths from cancer occurred in 10.2%, a percentage double than that reported in a study from a CVID cohort in New York over the same length of time (2, 9). We suggested that the discrepancy in cancer survival, between the two cohorts, might have been due to the high prevalence of deaths for malignancies other than lymphoma in the Italian CVID cohort, and to deaths for gastric cancer. The percentage of patients who died for lymphoma, indeed, was similar in the two studies.

Herein, we analyzed data on the prevalence of hematological and non-hematological malignancies, on cancer risk, on mortality and on survival rate in a cohort of 455 Italian adult CVID patients compared to normative population. Detailed data on CVID patients diagnosed with gastric cancer, histopathology of gastric lesions, cancer outcome and possible associated risk-factors were reported. Based on the high rate of mortality for gastric cancer in Italian CVID patients, we highlight the need of a strategy for an earlier diagnosis and we suggest a new schedule for gastric endoscopy in CVID patients.

## Methods

### Study design

Data on adult CVID patients (>18 years old), regularly followed in three University-based PID referral centers located in Central Italy (Rome), Southern Italy (Naples), and Northern Italy (Padua-Treviso) were prospectively collected from 01/01/2001 to 31/12/2017 and retrospectively collected from 01/01/1993 to 31/12/2000. To be considered for analysis, subjects needed to fulfill the 2016 ESID revised criteria (http://esid.org/Working-Parties/Registry/Diagnosis-criteria). A set of variables was recorded for each patient including: gender, date of birth, date of CVID diagnosis, data on cancer diagnosis and histology, date of last follow up visit, vital status information, date and cause of death, CVID-associated diseases (infections, cancer, autoimmunity, unexplained persistent proliferation, and unexplained persistent enteropathy) and *Helicobacter pylori (H. pylori)* status. We excluded from the analysis patients whose data on date cancer occurrence and its outcome and on date of cancer diagnosis, death and last follow-up were lacking. The follow-up period before the occurrence of cancer was calculated since the year of immunodeficiency onset. All subjects were followed until date of death or date of the end of the study (31 December 2017). For the subset of patients who developed cancer, medical records were traced to verify cancer diagnosis, treatments received, clinical complications, and outcome.

### AIRTUM estimated cancer incidence

The Associazione Italiana Registro Tumori (AIRTUM) (www.registri-tumori.it) is a coordinated system of population-based cancer registries that collects cancer incidence and survival data from 20 geographic areas throughout Italy, covering 70% of the Italian population (without age restriction). Detailed information is available at http://www.registri-tumori.it/. We used AIRTUM published data to estimate the expected incidence of cancer. Among skin malignancies, melanoma was the only one cancer with data on incidence and mortality reported in the AIRTUM database. For this reason, we did not collected data for not-malignant skin cancer.

### Statistical analysis

Demographics of the CVID database were summarized with descriptive statistics. Sociodemographic and clinical variables were compared between the patients who developed cancer and cancer-free patients. Statistical analysis was performed using frequency distributions. The *X*^*2*^ test was used for categorical variables and the *t-test* was used for continuous variables. The observed numbers of cancer cases among CVID were compared with the expected numbers calculated based on AIRTUM data on incidence rates of cancer in 5-year interval to yield the standardized incidence ratio (SIR). “All cancer” and site-specific cancer SIRs were calculated for the entire cohort, and separately for men and women. For mortality analysis, the time since diagnosis was determined using the age at the time of CVID diagnosis or the age at birth. The endpoint used was the time of last known follow-up or the date of death. Probabilities of survival after the diagnosis of CVID and after the diagnosis of cancer were estimated from Kaplan Meier life Table. Mortality rates (crude death rates, CDRs) of the general population were used to calculate the standardized mortality ratio (SMRs). The CDR was obtained from AIRTUM. SMRs were calculated using the formula, SMR = Observed (Obs) deaths/expected (Exp) deaths. We calculated SMRs as incident cases divided by the contributed person-years. However, general population incidence and mortality data for Italy before 2003 were not available, so only cancer and death occurred after 2003 were included in the analysis. Statistical Package for Social Sciences version 15 (SPSS Inc., 233 South Wacker Drive, 11th Floor, Chicago) was used for the analysis. Confidence Intervals (CI95%) were calculated by R-3.4.4 version.

## Results

### Patients

As of 31 December 2017, 501 subjects with a CVID diagnosis were included in the dataset. We excluded from the analysis 46 subjects who did not satisfy ESID criteria and patients whose date of death, date of cancer occurrence and outcome, and date of last follow-up could not be accurately determined. Data on 455 CIVD patients were included in the analysis. The characteristics of CVID patients enrolled in the study are summarized in Table 1. The mean age at last follow-up was 51.1 ± 15.0 years, with a 1:1 female: male ratio. Patients were followed-up for a cumulative period of 5,169 person-years with a mean time of follow-up of 11.5 ± 8.9 years. *H. pylori* status was available in 325/455 patients. *H. pylori* infection by histology was found in 40 patients (12%).

**Table 1:** Characteristics of 455 CVID patients enrolled in the study.

**Characteristics**	**All patients**	**Cancer**	**Cancer-free**
**AGE INTERVAL—*****n*****. (SD)**			
18-35 years	75 (16.5)	7 (6.0)	68 (20.0)^***^
36–50 years	142 (31.2)	29 (25.0)	113 (33.3)
51–65 years	150 (32.9)	45(38.8)	105 (31.0)
66–80 years	82 (18.0)	33 (28.5)	49 (14.5)^**^
>80 years	6 (1.3)	2 (1.7)	4 (1.2)
Sex (female)—*n*. (%)	235 (51.6)	58 (50.0)	162 (47.8)
Age at CVID diagnosis—mean (SD)	40.1 (15.4)	45.8 (13.2)	38.8 (15.7)^***^
**SERUM IMMUNOGLOBULIN AT DIAGNOSIS (mg/dL)—MEAN (SD)**			
IgG	250.3 (172.3)	256.2 (168.6)	248.7 (173.6)
IgA	21.6 (34.2)	19.9 (30.8)	22.1 (35.1)
IgM	25.2 (49.8)	37.0 (89.8)	21.9 (30.4)^*^
Bronchiectasis	118 (31)	26 (31)	92 (30)
Autoimmunity	130 (28)	27 (28)	103 (29)
Lymphoproliferative	113 (31)	28 (33)	85 (29)
Enteropathy	52 (14)	11 (14)	41 (14)
Time of follow-up person-year—mean (SD)	11.5 (8.9)	11.8 (8.4)	11.4 (9.2)
Patients with cancer—*n*. (%)	116 (25.5)	–	–
Patients with more than one cancer—*n*. (%)	18 (4.0)	–	18(15.5)
Patients alive at the last follow up—*n*. (%)	377 (82.9)	51(44.0)	27 (8.0)

* p < 0.01,

** p < 0.001, and

*** p < 0.0001 (cancer vs. cancer-free CVID patients).

*SD, standard deviation; CVID, common variable immunodeficiency; Ig, immunoglobulin. Immunoglobulin normal range (adults): IgG 700–1600 mg/dL; IgA 68–400 mg/dL; IgM 40–259 mg/dL*.

### Cancer prevalence and risk in CVID patients

During the study time, 132 separate cancers were diagnosed in 116 patients (25.5%). Eighteen patients (4%) developed more than one cancer. The age at CVID onset was higher for patients who developed cancer in comparison to those who did not (45.9 ± 13.2 vs. 38.1 ± 15.7 yrs, *p* < 0.0001, Table 1). The mean age at the first cancer diagnosis was 52.5 ± 13.8 (range: 26–85 yrs). Sixty-seven cancers were diagnosed in women and 65 cancers in men. Malignancies diagnosed were: lymphoma (38; 29%), gastrointestinal cancers (35; 26%), genitourinary cancers (14; 8%), breast cancers (10; 7%), uterine cancers (7; 5.3%), thyroid cancers (6; 4%), lung cancers (4; 3%), liver cancers (4; 3%), prostatic cancer (3; 2%), and pancreatic cancer (3; 2%). The overall and sex-related prevalence of single cancer was summarized in Table 2.

**Table 2:** Prevalence of cancer diagnosis in 455 Italian CVID patients.

**Cancer diagnosis—*n*. %**	**All patients** ***n*****. 455**		**Female** ***n*****. 235**		**Male** ***n*****. 220**	
	***n***.	**%**	***n***.	**%**	***n***.	**%**
Non-Hodgkin lymphoma	33	7.3	15	6.4	18	8.2
Gastric cancer	25	5.5	9	3.8	16	7.1
Colorectal cancer	10	2.2	4	1.7	6	2.7
Breast cancer	10	2.2	10	4.3	-	-
Thyroid cancer	6	1.3	3	1.3	3	1.3
Hodgkin lymphoma	5	1.1	2	0.9	3	1.3
Large Granular Lymphocytic Leukemia	5	1.1	3	1.3	2	0.9
Lung cancer	4	0.9	2	0.9	2	0.9
Liver cancer	4	0.9	2	0.9	2	0.9
Uterine cancer, body	4	0.9	4	1.7	–	–
Uterine cancer, cervical	3	0.7	3	1.3	–	–
Prostatic cancer	3	0.7	–	–	3	1.3
Pancreatic cancer	3	0.7	2	0.9	1	0.4
Other blood cancer (CML, polycythemia vera)	3	0.7	1	0.4	2	0.9
Kaposi sarcoma	1	0.2	0	–	1	0.4
Others^#x0002A;^	13	3.5	7	3.0	6	2.7

* *Others: Bladder cancer, meningioma, melanoma, neuro-endocrine carcinoma, ocular carcinoma, kidney carcinoma, adrenal carcinoma*.

**Figure 1:**
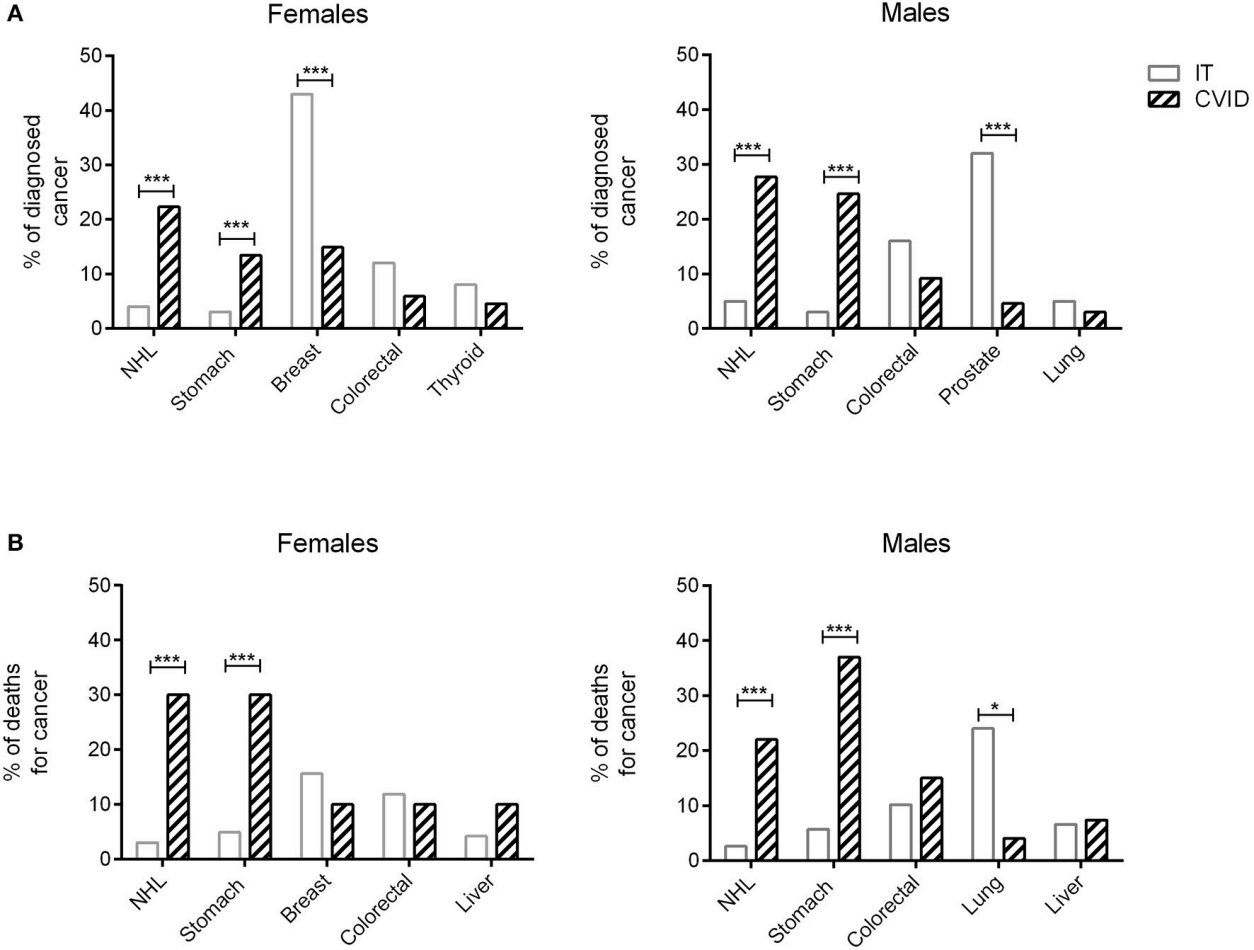
Cancers diagnosis and death for cancer in CVID and in the normative population. Data related to the proportion of the five most frequently diagnosed cancers in male and female CVID patients (dashed bars) are shown in comparison to the normative population (IT, white bars) **(A)**. Proportion of deaths for cancer in male and female CVID patients (dashed bars) are shown in comparison to the normative population (IT, white bars) **(B)**. In CVID, NHL and gastric cancer were the most commonly diagnosed cancers in both sexes, whereas breast cancer and prostate cancer were the most frequently recorded malignancies in Italian normative population. Gastric cancer was the first cause of death for cancer in CVID females and males, followed by NHL; breast and lung cancers were the most common cause of death for cancer in normative population. Data of normative population referred to 2017 AIRTUM report. NHL, non-Hodgkin lymphoma. ^*^p < 0.01; ^***^p < 0.0001.

Figures 1A,B showed the percentage of the top five diagnosed cancers and the top five fatal cancers seen in CVID in comparison to the normative Italian population. The most common malignancy diagnosed in the AIRTUM database was breast cancer (women only), prostate cancer (men only), lung, and colorectal cancers. The incidence of these cancers was not increased in CVID patients in comparison to the AIRTUM database (Table 3). Ten female CVID patients were diagnosed with breast cancer (Exp: 10.0; SIR: 1.0; 95%CI: 0.7–1.2). Three male CVID patients were diagnosed with prostate cancer (Exp: 7.1; SIR: 0.4, 95%CI: 0.1–1.0). There was no increase in the rates of lung cancer (Obs: 4, Exp: 28.0, SIR 0.1; 95%CI: 0.2–0.7) and colon cancer (Obs: 10; Exp: 8.2; SIR 1.2; 95%CI: 0.0–1.9) among CVID patients vs. normative population (Table 3). In contrast to Italian normative population, the most commonly diagnosed malignancies in female and male subjects with CVID were non-Hodgkin lymphoma (NHL) and gastric cancer (Figure 1A). The risk for NHL and Hodgkin's lymphoma (HD) was increased by 14.3- and 12.5-fold, respectively, based on 33 and 5 cases observed. The risk for gastric cancer was increased 6.4-fold based on 25 cases observed (Table 3).

**Table 3:** Observed (Obs) and Expected (Exp) numbers and Standardized Incidence Ratio (SIR) of cancer among 455 Italian patients with CVID.

	**Obs**	**Exp**	**SIR**	**95%CI**
**CANCER**				
All malignant neoplasms	133	55.1	2.4	1.7–3.5
Non-Hodgkin lymphoma	33	2.3	14.3	8.4–22.6
Gastric cancer	25	3.9	6.4	3.2–12.5
Colorectal cancer	10	8.2	1.2	0.0–1.9
Breast cancer	10	10	1	0.7–1.2
Thyroid cancer	5	1.7	2.9	0.0–6.4
Hodgkin Disease	5	0.4	12.5	3.4–22.4
Lung cancer	4	28	0.1	0.2–0.7
Liver cancer	4	2.1	1.9	0.3–5.6
Uterine cancer, body	4	1.2	3.3	0.1–6.5
Uterine cancer, cervical	3	1.2	2.5	0.1–4.8
Prostatic cancer	3	7.1	0.4	0.1–1.0
Pancreatic cancer	3	1.9	1.6	0.3–3.9

### Survival and mortality

**Figure 2:**
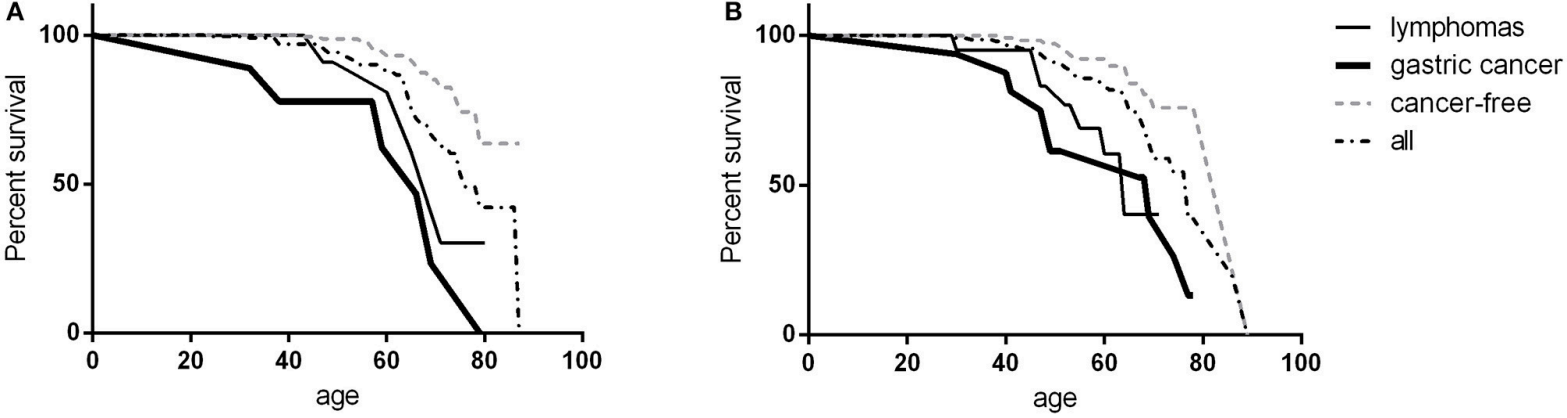
CVID survival. Survival in female **(A)** and male **(B)** CVID participants: data were shown as overall survival (black dashed line), in CVID patients with gastric cancer (black bold line), in patients with lymphoma (black line) and in cancer-free CVID patients (gray dashed line). No survival differences were observed between females and males; CVID subjects with gastric cancer or lymphoma had a worse survival in comparison to cancer-free CVID population.

Three-hundred and seventy-four (82.4%) patients were alive at the end of the study-time. During the study time, we observed 78 deaths in the patient population. Malignancies were the first cause of death, accounting for 60.3% of deaths. Gastric cancer was the leading cause of death (20.5%). Infections accounted for 19.2% of deaths, 80% due to lower tract respiratory infections. Causes of mortality in patients are detailed in Table 4. The CVID overall survival (OS) was 85.1% (SE 2.3%) at 60 years and 61.8% (SE 4.2%) at 70 years. No differences were observed between males and females. Cancer-free CVID had a better survival in comparison to those with gastric cancer (Log-Rank *p* < 0.0001, HR: 4.8, 95%CI: 4.2–44.4, Figure 2A) and lymphomas (Log-Rank *p* = 0.001, HR: 4.2, 95%CI: 2.8–44.4, Figure 2B).

**Table 4:** Cause of death in CVID patients.

**Causes of death**	***n***.	**%**
Cancer	47	60.3
Gastric cancer	16	20.5
Non-Hodgkin Lymphoma	14	17.9
Colorectal cancer	6	7.7
Liver cancer	4	5.1
Pancreatic cancer	2	2.6
Breast cancer	2	2.6
Hodgkin Disease	1	1.3
Lung cancer	1	1.3
Uterine cancer	1	1.3
Infections	15	19.2
LRTI (respiratory failure)	12	10.3
Other infections (sepsis, CMV)	3	10.3
Cardiovascular disease	5	6.4
Autoimmune manifestations: AHA, AIH	4	5.1
Others^*^	7	9.0
Total	78	–

LRTI, lower respiratory tract infections; AHA, autoimmune hemolytic anemia; AIH, autoimmune hepatitis.

* *Parkinson disease, cirrhosis, accident, suicide*.

Cancer excess of mortality was expressed as SMRs (Table 5). The most fatal cancers in the AIRTUM database were lung, colorectal and breast cancer. We found no significant increase in the mortality for colorectal cancer (Obs: 6; Exp: 2.1; SMR: 2.8, 95%CI: 0.1–6.3) and a significant lower mortality for lung and breast cancers in CVID patients (lung cancer: Obs: 1; Exp: 6.6; SMR: 0.1; 95%CI: 0.3–0.5; breast cancer: Obs; 2; Exp: 7.0; SMR: 0.3; 95%CI: 0.0–0.5, Table 5). Moreover, in CVID we found an excess of mortality for NHL (Obs: 14; Exp: 0.8; SMR: 16.5; 95%CI: 8.8–31.4) and gastric cancer (Obs: 16; Exp: 2.0; SMR: 10.1; 95%CI: 3.8–16.3) (Table 5).

**Table 5:** Standardized mortality ratios (SMRs) for cancers causing death in CVID.

**Cancers**	**Obs**	**Exp**	**SMR**	**95%CI**
All malignant neoplasm	47	44.5	1.0	0.5–1.6
Gastric cancer	16	2.0	10.1	3.8–16.3
Non-Hodgkin lymphoma	14	0.8	16.5	8.8–31.4
Colorectal cancer	6	2.1	2.8	0.1–6.3
Liver cancer	4	1.8	2.9	0.1–5.9
Pancreatic cancer	2	1.7	1.2	0.6–3.2
Breast cancer	2	7.0	0.3	0.0–0.5
Hodgkin Disease	1	0.1	10.0	0.0–45.2
Lung cancer	1	6.6	0.1	0.3–0.5
Uterine cancer	1	0.3	2.8	0.0–8.3

### Gastric cancer

**Figure 3:**
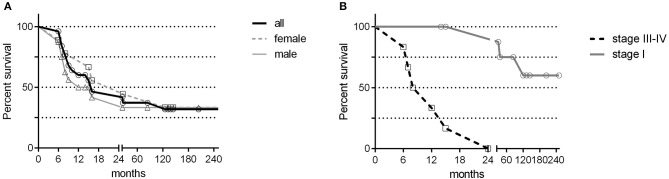
Gastric cancer survival by sex and staging. Survival in the cancer free CVID subjects (black bold line) and in CVID females (gray dashed line) and males (gray line) with gastric cancer was shown in **(A)**. Survival in patients scored as stage I (gray line) and in patients scored as stage III-IV (dashed line) was shown in **(B)**. No difference was observed between CVID females and males with gastric cancer; patients scored as stage I had a better survival in comparison to patients scored as stage III-IV.

Gastric cancer was the second most frequent cancer diagnosed in CVID accounting for 18.9% of cancer diagnosed, and the first cause of death. Of the 25 cases of gastric cancer, 16 (64%) occurred in men. The age at cancer diagnosis was 51.8 ± 13.7 years (range: 30–75), 15 years younger than that reported in the Italian population (10). The diagnosis of cancer occurred within 10 years from the CVID diagnosis in two thirds of these patients. CVID subjects with gastric cancer had a similar age at immunodeficiency onset than the entire CVID cohort (40.0 ± 15.0 vs.39.9 ± 15.4, *p* = 0.975). Undetectable IgA (<7 mg/dL) and IgM (<6 mg/dL) serum levels at time of CVID diagnosis were more likely in patients with gastric cancer in comparison to those without that complication (IgA: OR 27.5, 95%CI: 1.5–475.9, *p* = 0.027; IgM: OR 7.4, 95% CI: 1.5–36.1, *p* = 0.013). Seven out of 25 patients had an additional malignancy: three patients were diagnosed with lymphoma, two with colorectal cancer, one with gallbladder cancer, and one with meningioma. In addition, two patients had a multifocal gastric adenocarcinoma treated by two-stage gastrectomy (Table 6). One patient (n. 1) had a positive family history for gastric cancer: three relatives, including his IgA-deficient brother, developed the malignancy. In these two brothers, mutations of *CDH1* gene were not found. Fifteen patients with gastric cancer died during the study time. Overall, the average SMR indicated a 10.1-fold excess mortality among CVID patients with gastric cancer. The 10-year survival probability of the entire cohort of patients with gastric cancer was 25%. Clinical staging was available for 16/25 patients. Patients classified as stage I had a better survival in comparison to those with stage III-IV (HR: 0.01, 95%CI: 0.0–0.1, *p* < 0.0001, Figure 3). *H. pylori* status and histology of gastric endoscopic biopsies collected before the diagnosis of cancer was available for 7 patients (Table 7): areas of dysplasia were identified in two subjects whereas areas of atrophic gastritis and/or intestinal metaplasia were found in all patients. In the two patients with dysplasia, the following endoscopy revealed a stage I malignancy, 6 months apart (patient n. 23). Patient n. 25 agreed to undergo a further gastroscopy only 15 months apart, which allowed diagnosis of a stage IV gastric cancer. Interesting to note, patient n. 20 and n. 22 developed a high-grade gastric cancer <14 months after the preceding endoscopy whose histology did not show any signs of dysplasia. At least one *H. pylori* detection was significantly related to gastric cancer (43 vs. 13%, OR: 5.3, 95%CI: 1.1–24.8, *p* = 0.042).

**Table 6:** Age at PID and at cancer diagnosis, survival, outcome, histology, cancer stage, and cancer treatment in 25 CVID patients with gastric cancer.

**ID**	**Sex**	**Age at PID diagnosis** **(years)**	**Age at cancer diagnosis** **(years)**	**Survival after cancer diagnosis** **(months)**	**Outcome**	**Histology**	**Stage**	**Treatment**	**Additional cancer**	**Enteropathy (before cancer diagnosis)**
1	M	39	40	408	Alive	Early gastric cancer, pT1N0 G1	stage I	Gastrectomy (total)	Kaposi sarcoma, colorectal carcinoma	No
2	F	25	31	12	Deceased (cancer)	Gastric adenocarcinoma, NOS	NA	Chemotherapy, NOS	No	Yes
3	F	45	45	252	Alive	Gastric adenocarcinoma, NOS	NA	Gastrectomy, NOS	Meningioma	No
4	F	67	69	120	Deceased (cancer)	Early gastric cancer pT1N0 G2,	stage I	Gastrectomy (total)	Biliary tract carcinoma	NA
5	M	40	49	7	Deceased (cancer)	Gastric adenocarcinoma, NOS	NA	Gastrectomy (total)	No	Yes
6	M	27	51	204	Alive	Gastric adenocarcinoma, intestinal type pT1N0 G2	stage I	Gastrectomy (total)	NHL (duodenal) lymphoma	No
7	M	58	74	36	Deceased (cancer)	Gastric adenocarcinoma, intestinal type, PT1, N0, M0	stage IA	Gastrectomy (subtotal)	No	Yes
8	F	35	45	144	Alive	Gastric adenocarcinoma, NOS pT1bN1 G1	stage IB	Gastrectomy (subtotal)	No	No
9	M	67	67	132	Alive	Gastric adenocarcinoma, NOS		Gastrectomy (subtotal)	Colorectal carcinoma	Yes
10	F	32	38	132	Alive	Early gastric cancer, pT1N0 G2	stage I	Gastrectomy (subtotal)	HD	Yes
11	M	64	67	30	Deceased (cancer)	Gastric adenocarcinoma, intestinal type, PT1, N0, Mx, G3	stage I	Gastrectomy (subtotal), Chemotherapy (lederfolin, xeloda, 5-fluorouracil)	No	Yes
12	M	69	75	24	Deceased (cancer)	Gastric adenocarcinoma, NOS	NA	Supportive	No	Yes
13	F	35	68	12	Deceased (cancer)	Gastric Adenocarcinoma, G3	NA	Supportive	NHL	Yes
14	F	27	38	9	Deceased (cancer)	Gastric adenocarcinoma, NOS	NA	Supportive	No	NA
15	M	30	47	12	Deceased (cancer)	Gastric adenocarcinoma, NOS	stage IIIB	Chemotherapy, NOS	No	Yes
16	M	40	40	84	Alive	Multifocal Gastric Adenocarcinoma pT2bN0 G3 CMV+	stage IB	Gastrectomy (total, two-step)	No	No
17	M	59	68	12	Deceased (cancer)	Gastric adenocarcinoma, NOS	NA	Supportive	No	Yes
18	M	36	48	11	Deceased (respiratory failure)	Gastric adenocarcinoma, NOS	NA	Chemotherapy, NOS	No	NA
19	M	18	40	15	Deceased (cancer)	Gastric adenocarcinoma, intestinal type pT3N3bMx G3	stage IIIC	Gastrectomy (total), Chemotherapy (platinum/5-fluorouracil)	No	Yes
20	F	43	64	24	Deceased (cachexia, meningitis)	Gastric adenocarcinoma, intestinal type pT3N2 G3	stage IIIA	Gastrectomy (subtotal), + Capecitabine	No	No
21	M	22	30	8	Deceased (cancer)	Gastric adenocarcinoma, NOS	stage IV	Supportive	No	No
22	M	29	40	7	Deceased (cancer)	Gastric adenocarcinoma, NOS	stage IV	Chemotherapy, NOS	No	Yes
23	M	49	51	15	Alive	Multifocal gastric adenocarcinoma, intestinal type, pT1pN0 G3	stage I	Gastrectomy (total, two-step)	No	No
24	M	47	50	14	Alive	Early gastric cancer, intestinal type, pT1bNx G3	stage I	Gastrectomy	No	No
25	F	46	59	6	Deceased (cancer)	Gastric adenocarcinoma G3	stage IV	Chemotherapy (epirubicine, platinum, 5-fluorouracil)	No	Yes

*NOS, not otherwise specified; NA, not available M, Males, F, Females; NHL, Non-Hodgkin lymphoma; HD, Hodgkin disease*.

**Table 7:** *H. pylori* status and histology of gastric biopsies from the endoscopy preceding the examination leading to gastric cancer diagnosis in seven CVID patients.

**ID**	***H. pylori* (pos/neg)**	**Histology of biopsy taken at the endoscopy preceding the one with gastric cancer diagnosis**	**Interval between endoscopies (months)**	**Stage at cancer diagnosis**	**Outcome**
9	pos	Active chronic gastritis, intestinal metaplasia	36	NA	Alive
12	neg	Atrophic gastritis	35	NA	Death
13	pos	Atrophic gastritis	12	NA	Death
20	neg	Active chronic gastritis (moderate) with incomplete intestinal metaplasia	14	Stage IIIA	Death
22	pos	Active chronic gastritis (moderate), intestinal metaplasia	14	Stage IV	Death
23	neg	Atrophic gastritis, high grade dysplasia	6	Stage I	Alive
25	neg	Intestinal metaplasia, high-grade dysplasia	15	Stage IV	Death

*NA, not available*.

### Lymphoma

Lymphoma was the most frequent cancer diagnosed in CVID and the second cause of death for cancer (Tables 2, 4). The age at lymphoma diagnosis was 32.8 ± 4.6 years for HD and 52.4 ± 13.1 years for NHL. The age at CVID diagnosis was higher in patients with lymphoma in comparison to those without lymphoma (45.7 ± 12.4 vs. 39.6 ± 15.6, *p* = 0.008). Three patients first presented with lymphoma prior to CVID diagnosis, raising the question if hypogammaglobulinemia might be secondary to the lymphoproliferative disease (Table 8). However, the longtime state of antibody defect after the diagnosis and treatment of lymphoma might suggest this possibility. As widely described (2–9, 11), also in our cohort, CVID-associated lymphoma was more likely to be of B cell origin (88.4%) with a predominance of NHL (81.8%). T-cell lymphomas (peripheral T-cell lymphoma, angio-immunoblastic T-cell lymphoma and anaplastic T-cell lymphoma) and one primitive effusion cavity (PEL) lymphoma were also observed (Table 8). Similar to what observed in other series of CVID patients about 30% were extra-nodal lymphomas. Patients with lymphoma were more likely to have lymphopenia (lymphocytes < 1,000 cell/mm^3^) (OR: 3.0, 95%CI: 1.1–8.3, *p* = 0.030) and polyclonal lymphocytic infiltration phenotype (OR: 2.7, 95%CI: 1.2–6.3, *p* = 0.016) before cancer diagnosis.

**Table 8:** Characteristics of CVID patients diagnosed with lymphoma.

**ID**	**Sex**	**Age at cancer onset**, **years**	**Age at CVID diagnosis, years**	**Survival after cancer, months**	**Outcome (cause)**	**Histology and stage**	**Treatment**	**Additional cancer**
6	M	50	37	216	Alive	Diffuse large B cell lymphoma (small bowel)	Chemotherapy NOS	Gastric cancer
10	F	32	42	336	Alive	HD	CHOP, ABV	Gastric cancer
13	F	67	35	12	Deceased (gastric cancer)	Diffuse large B cell lymphoma	NA	Gastric cancer
26	F	47	47	8	Deceased (lymphoma)	NHL not further classified	NA	No
27	M	38	37	120	Alive	Diffuse large B cell lymphoma of small bowel, stage IVE	R-CHOP	No
28	F	50	44	13	Deceased (lymphoma)	T-cell lymphoma (peripheral T cell lymphoma)	CHOP, autologous HSCT, Brentuximab	No
29	F	64	58	12	Deceased (lymphoma)	T-cell lymphoma (angioimmunoblastic T cell lymphoma)	Prednisone	No
30	M	40	47	144	Deceased (lymphoma)	NHL not further classified	NA	No
31	M	48	41	132	Alive	NHL not further classified	NA	No
32	F	74	47	24	Alive	Diffuse large B cell lymphoma (large bowel)	R-CHOP, RTX	No
33	M	53	47	9	Alive	Diffuse large B cell lymphoma (T-cells rich)	R-CHOP	No
34	M	29	30	14	Alive	Diffuse large B cell lymphoma	R-CHOP	No
35	M	58	55	36	Alive	NHL not further classified	Chemotherapy NOS	No
36	M	59	35	12	Deceased (lymphoma)	Cutaneous diffuse large B cell lymphoma leg-type	R-CHOP, radiotherapy	No
37	F	67	43	84	Alive	Marginal Zone Lymphoma (Splenic)	Splenectomy	Lung cancer
38	M	54	47	84	Alive	NHL not further classified	NA	No
39	F	29	28	24	Alive	Lymphoplasmacytic lymphoma	Chemotherapy NOS	No
40	M	47	45	6	Deceased (lymphoma)	NHL not further classified	NA	No
41	F	65	62	8	Deceased (lymphoma)	Diffuse large B cell lymphoma	NA	No
42	F	62	56	12	Alive	Marginal Zone Lymphoma (nodal and extra nodal)	RTX, bendamustine	No
43	M	60	60	36	Alive	Marginal Zone Lymphoma (nodal and extra nodal)	R-CHOP	No
44	M	47	47	72	Alive	Anaplastic T cell Lymphoma ALK- stage IVB (skin)	CHOEP, FEAM and autologous HSCT	No
45	F	67	64	11	Deceased (lymphoma)	NHL not further classified	NA	No
46	F	61	70	168	Deceased (cardiovascular)	Diffuse large B cell lymphoma (small bowel)	Ileocecal resection + R-CHOP	No
47	F	41	38	24	Alive	Marginal Zone Lymphoma	NA	No
48	M	52	56	120	Alive	NHL, not further classified, stage IV	R-FN, R-CHOP	No
49	M	70	59	12	Alive	Marginal Zone Lymphoma (nodal, indolent behavior)	Rituximab + bendamustine	Prostatic cancer
50	M	41	40	15	Alive	Kaposi sarcoma/Primitive effusion lymphoma, HHV8+/EBV+	CDE	No
51	F	74	62	72	Alive	MALT Lymphoma (gastric)	NA	No
52	M	59	59	60	Deceased (lymphoma)	Diffuse large B cell lymphoma (lung), stage IVB	R-CHOP, R-COMP	No
53	F	45	54	216	Deceased (lymphoma)	NHL not further classified (low grade)	R-CHOP + Etoposide	Uterine cancer, body
54	M	55	55	10	Deceased (lymphoma)	NHL not further classified	NA	No
55	M	36	21	108	Alive	HD, classic type, stage IIIsB	ABVD	No
56	M	30	28	12	Deceased (lymphoma)	HD, classical type, lymphocyte-depleted, stage IV	Chemotherapy NOS	Thyroid cancer
57	F	28	38	12	Deceased (T-cell lymphoma)	HD, mixed-cellularity type	Radiotherapy	Thyroid cancer, angio-immunoblastic T cell lymphoma)
58	M	29	18	8	Alive	HD, sclero-nodular type	ABVD, radiotherapy	No
59	M	39	38	96	Deceased (lymphoma)	HD, classical type, stage IVA	VEBEP, HSCT	No

*ABV, Adriamycin, Hydroxydaunorubicin, Bleomycin, Vinblastine; ABVD, Adriamycin, Hydroxydaunorubicin, Bleomycin, Vinblastine, Dacarbazine; CHOEP, Cyclophosphamide, Doxorubicin, Etoposide, Vincristine, Prednisone; CHOP, Cyclophosphamide, Hydroxydaunorubicin, Vincristine, Prednisone; CDE, Cyclophosphamide, Doxorubicin, Etoposide; COPP, Cyclophosphamide, Vincristine, Procarbazine, Prednisone; FEAM, Fotemustine, Etoposide, Cytarabine, Melphalan; R-CHOP, Rituximab, Cyclophosphamide, Hydroxydaunorubicin, Vincristine, Prednisone; FN-R, Rituximab, Fludarabine, Mitoxantrone; HD, Hodgkin disease, NHL, Non-Hodgkin lymphoma, HSCT, Hematopoietic stem cell transplantation; R-COMP, Rituximab, Cyclophosphamide, Vincristine, Myocet, Prednisone; RTX, Rituximab; VEBEP, Etoposide, Epirubucin, Bleomycin, Cyclophosphamide, Prednisolone; NOS, not otherwise specified; NA, not available*.

## Discussion

This longitudinal study on a large cohort of CVID patients over a cumulative period of 5,169 person-years showed that one fourth of patients developed a malignancy. Cancer represents the first cause of death in our patient's population. The most commonly diagnosed malignancies in CVID were NHL and the first cause of death was gastric cancer. The excess of mortality for lymphoma and gastric carcinoma in CVID was increased by more than 10-fold in comparison to normative population. Several studies reported a high frequency of malignancies in CVID patients (2, 3, 6–9, 11–14) with a prevalence ranging from 1.5 to 20.7%. However, only few studies provided SIR, allowing the comparison of data on CVID to data on normative population. These surveys showed an excess of incidence ranging from 4- to 30-fold for NHL and from 3- to 47-fold for gastric cancer (3, 6, 7, 15). Nevertheless, the prevalence other malignancies was not increased, confirming that patients with antibody deficiencies have a narrow range of cancers (6).

Lymphoma is considered as one of the more severe complications of CVID. The prevalence registered in our cohort was similar to that found across the different countries examined (2–9, 11, 12). The histological types reported in the different series of CVID-associated lymphoma were also similar to our findings, with a predominance of non-Hodgkin B cell lymphomas, possibly occurring at extra nodal sites. We confirmed the observation by Chapel et al. showing that CVID patients with polyclonal lymphadenopathy phenotype have an increased risk of lymphoid malignancy that generally occurs late in the disease course (16). In addition, we found that CVID patients with lymphopenia had a 3-fold increased risk to develop lymphomas. In CVID patients, diagnosis of lymphoma may be particularly challenging. Immune-histochemical analysis, studies on clonality and molecular studies might be helpful to distinguish reactive from neoplastic lymphoproliferative diseases, even if CVID patients with clonal B cell expansion, who survive without developing an overt lymphoma, have been described (12, 17). Treatment of CVID-associated lymphoma was usually like the treatment of lymphoma in other settings and usually it included rituximab.

Herein, to the best of our knowledge, we collected the largest case-series of gastric cancers in CVID subjects ever described, showing a high prevalence and an excess of mortality for gastric cancer. However, the SIR for gastric cancer was similar to that found across other studies providing this kind of figure (3, 6, 7). This difference might be related to the observation that gastric cancer prevalence may vary significantly within and between countries (18). In comparison to the normative population, CVID patients were on the average 15-years younger at the time of cancer onset. As reported for non-CVID subjects (19), CVID patients with early-stages gastric cancer had a better prognosis in comparison to those with more advanced stage, who died within 2 years since cancer diagnosis. According to our data, chronic atrophic gastritis and extensive intestinal metaplasia are invariably associated with gastric cancer in CVID. Similarly, De Petris et al. (20) showed that these adenocarcinomas were diagnosed at a young age and were of intestinal type. They were also associated with increased numbers of intra-tumoral lymphocytes, paucity of plasma cells and nodular lymphoid hyperplasia, all features suggestive of chronic inflammation of the gastric mucosa.

These observations gave us the chance to suggest the implementation of current screening strategy, aimed to an early diagnosis. The appropriate timing of upper endoscopy in CVID is a matter of debate. In the general population, Rugge et al. suggested performing upper endoscopy every 2 years in subjects with gastritis scored as stage III–IV (10). In CVID, Dhalla et al. (21) suggested to perform upper endoscopy in patients with risk factors for gastric cancer (*H. pylori* positivity, low serum vitamin B12 and iron concentrations) with an interval between the subsequent endoscopic assessment based on histological findings: every 1–3 years in CVID patients with metaplasia, every 3 years in patients with atrophic gastritis, and every 6–12 months in those with dysplasia. However, this interval may not be suitable for CVID, who rapidly develop advanced-stage cancer with poor prognosis. In fact, we showed that some CVID developed a high-grade gastric cancer already 12–14 months after an endoscopy showing no histologic signs of dysplasia. This rapid cancer development in CVID was unexpected since no epithelial gastric cancer was identified in patients without signs of dysplasia on a cohort of 1,615 Italian non-CVID followed over a 1–5 years period (10).

*H. pylori* eradication represents the main strategy to reduce the lifetime risk of gastric cancer since *H. pylori* is widely recognized as the leading cause of gastric cancer (22). In CVID, serological tests are not useful to identify *H. pylori* positive patients and only direct diagnostic methods for *H. pylori* detection should be considered (23). However, follow-up strategies targeted at gastric cancer secondary prevention cannot rely only on *H. pylori* identification, since the eradication of *H. pylori* might not abolish the risk for neoplastic progression (24–26). This is supported by our observation in two CVID patients who developed gastric cancer 1–2 years after a *H. pylori*-negative gastric biopsy.

On the basis of our data, we recommend the implementation of national guidelines based on regular upper endoscopy and on treatment of *H. pylori* infection. We propose to always perform upper endoscopy at the time of CVID diagnosis; to repeat endoscopy every 24 months in patients with normal histology; every 12 months in patients with atrophic gastritis or intestinal metaplasia, and every 6 months in patients with dysplastic lesions. Diagnosis of *H. pylori* infection should be actively ruled out at diagnosis and during the course of CVID disease. The prevalence and the risk of gastric cancer detected in Italian CVID patients might be related to the epidemiology of *H. pylori* infection in our country. Thus, further studies should be undertaken in other countries before they adopt our suggested measures of disease management. However, a careful endoscopic monitoring of gastric cancer should be advisable also in countries with low *H. pylori* prevalence, since the rate of antibiotic-resistant strains is increasing worldwide (22).

Our study has some limitations. First of all, we included in the analysis also retrospective data with possible survival bias. Second, we did not include in the analysis the genetic diagnoses of the cohort. Since it has been shown the risk of gastric cancer was not increased among relatives of CVID patients (7), however, it is possible that cancer morbidity might be related to the immunodeficiency *per se* rather than to family habits or environmental factors, including *H. pylori* sharing. Finally, preliminary data suggested spontaneous gastric cancer in models of NFkappaB1 deficiency (27) and recent papers suggested that significant proportion of CVID patients may harbor haploinsufficient *NFKB1* mutations (28). Additional studies on alterations of gastric mucosal immunity and microbiota and on genetic alterations are needed to better understand the gastric carcinogenesis in CVID patients.

## Ethics statement

This study was carried out in accordance with the Good Clinical Practice guidelines, the International Conference on Harmonization guidelines, and the most recent version of the Declaration of Helsinki. The protocol was approved by Ethics Committee of Sapienza University of Rome and Azienda Policlinico Umberto I: Protocollo di osservazionale retrospettivo-prospettico sui soggetti affetti da Immunodeficienza Comune Variabile arruolati nei centri AIEOP/IPINET. Rif. CE:4063 on 04/14/2016.

## Author contributions

FP, GS, CA, and IQ: conceived and designed the study; FP, AP, FC, ES, LC, FR, and CM data collection; FP, CM, GS, CA, MV, ST, and IQ: data analysis and interpretation; FP, CM, GS, CA, ST, and IQ: manuscript preparation.

### Conflict of interest statement

The authors declare that the research was conducted in the absence of any commercial or financial relationships that could be construed as a potential conflict of interest.
